# Contribution of oxidative stress to endothelial dysfunction in hereditary hemorrhagic telangiectasia

**DOI:** 10.3389/fgene.2015.00034

**Published:** 2015-02-13

**Authors:** Mirjana Jerkic, Michelle Letarte

**Affiliations:** ^1^Anesthesia Research, Keenan Research Centre for Biomedical Science, St. Michael’s Hospital, University of Toronto, Toronto, ON, Canada; ^2^Molecular Structure and Function Program, Peter Gilgan Centre for Research and Learning, Hospital for Sick Children, University of Toronto, Toronto, ON, Canada; ^3^Department of Immunology, University of Toronto, Toronto, ON, Canada

**Keywords:** HHT, reactive oxygen species, nitric oxide, eNOS, antioxidants

## Abstract

Oxidative stress causes endothelial dysfunction and is implicated in the pathogenesis of cardiovascular diseases. Our studies suggested that reactive oxygen species (ROS) play a crucial role in hereditary hemorrhagic telangiectasia (HHT) disease, a vascular dysplasia affecting 1 in 5,000–8,000 people. Mutations in endoglin (*ENG*) and activin receptor-like kinase 1 (*ACVRL1*) genes are responsible for HHT1 and HHT2 and are associated with arteriovenous malformations. ENG and ACVRL1 interact with endothelial nitric oxide synthase (eNOS) and regulate its activation. Mice heterozygous for these genes (*Eng*^+/–^ and *Acvrl1*^+/–^) show reduced ENG or ACVRL1 protein levels in endothelial cells causing eNOS uncoupling, generation of ROS rather than nitric oxide (NO•), leading to impaired NO• mediated vasodilation. ROS production is increased in several organs of *Eng*^+/–^ and *Acvrl1*^+/–^ mice, including lungs, liver, and colon, affected in HHT. The major source of increased oxidative stress in these tissues is eNOS-derived ROS and not mitochondrial or NADPH oxidase-dependent ROS. *Eng*^+/–^ and *Acvrl1*^+/–^ mice also develop with age signs of pulmonary arterial hypertension attributable to eNOS-derived ROS, which was preventable by antioxidant treatment. To date, only one pilot study has been carried out in HHT patients, and it showed beneficial effects of antioxidant therapy on epistaxis. We suggest that more clinical studies are warranted to investigate whether antioxidants would prevent, delay or attenuate manifestations of disease in individuals with HHT, based on our experimental data in mouse models.

## INCREASED ROS PRODUCTION AND ENDOTHELIAL DYSFUNCTION IN CARDIOVASCULAR DISEASES

Reactive oxygen species (ROS) are important regulators of many processes including intracellular signaling, response to growth factor stimulation and generation of the inflammatory response (Finkel, 2011). Dysregulated ROS signaling, leading to increased free radical production and/or down regulation of ROS scavengers and antioxidants, may cause or accelerate many pathological conditions, including cardiovascular, neurodegenerative, and malignant diseases. Oxidative stress is associated with endothelial dysfunction in cardiovascular disorders such as systemic and pulmonary hypertension, atherosclerosis, and diabetes (Münzel et al., 2010). Understanding the mechanisms of excessive ROS production and their role in endothelial damage may provide opportunities for pharmacological intervention.

The major enzymatic sources of ROS in endothelial cells are mitochondrial respiratory enzymes, NADPH oxidases and uncoupled endothelial nitric oxide synthase (eNOS). It is important to mention that increased ROS production together with decreased nitric oxide (NO•) bioavailability can further complicate and worsen endothelial injury (Montezano and Touyz, 2012a). It is also recognized that crosstalk between various sources of ROS exists and may represent a feed-forward vicious cycle of ROS production (Dikalov, 2011).

**FIGURE 1:**
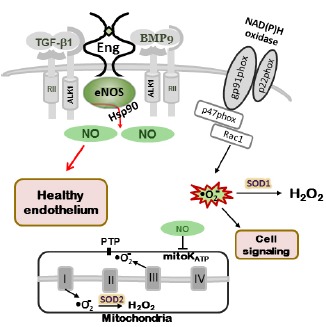
**NO production in normal endothelium.** In wild type mice (*Eng*^+/+^ and *Alk1*^+/+^) eNOS is closely associated with caveolae/lipid rafts in endothelial cells. Endoglin and ALK-1 co-localize with eNOS serving as scaffolding molecules for eNOS/Hsp90 association and eNOS activation, leading to NO• production. Normal cellular NO• levels through mitochondrial ATP-sensitive potassium channels (mitoK ATP) and closed permeability transition pores (PTP) prevent mitochondrial ROS “leak” into the cytoplasm. Small amount of ROS, produced by NADPH oxidases, are involved in cell signaling (adapted from Jerkic et al., 2012).

Mitochondria produce ROS during ATP generation, when electrons transferred through the respiratory chain “leak” and create superoxide (•O_2_^–^; Widlansky and Gutterman, 2011), which can then be scavenged by the superoxide dismutase 2 (SOD2) present in the mitochondrial matrix. The release of ROS depends on activation of mitochondrial ATP-sensitive potassium channels (mitoK ATP) and opening of the permeability transition pores (PTP). Interestingly, NO• affects mitoK ATP activation (Figure 1) and low levels induce mitochondrial ROS release causing additional cellular oxidative stress (Chang et al., 2010). Excessive production of mitochondrial ROS can be targeted by specific inhibitors of the electron respiratory chain, such as antimycin A (Kudin et al., 2008) or by increased ROS scavenging. Recent animal studies have demonstrated that overexpression of SOD2 reduces fibrosis and pro-apoptotic signaling in the aging mouse heart (Kwak et al., 2014), while MitoTEMPOL, an SOD mimetic drug that concentrates in mitochondria, significantly reduced H_2_O_2_- and lipid peroxide-induced endothelial oxidative stress (Dhanasekaran et al., 2005).

NADPH oxidases are a family of enzyme complexes, which catalyze the transfer of electrons from NADPH to molecular O_2_ via their “Nox” catalytic subunit, generating •O_2_^–^ and H_2_O_2_ (Dikalov, 2011; Figure 1). The major Nox isoforms present in endothelial cells are Nox4 and Nox5. ROS generated from NADPH oxidases also affect redox-sensitive mitoK ATP activation, since inhibition of NADPH oxidases by apocynin completely prevented mitochondrial dysfunction induced by Angiotensin II (AngII; Doughan et al., 2008). NADPH oxidases also appear to be a major source of ROS in vessels of spontaneously hypertensive rats, as demonstrated by blocking with the novel and more specific NADPH oxidase inhibitor VAS3947 (Wind et al., 2010). Studies on arteries from patients with coronary artery disease and in animals with experimentally induced systemic hypertension, diabetes, or atherosclerosis, suggest that Nox1, Nox2, and Nox5 promote endothelial dysfunction (Drummond and Sobey, 2014, whereas contribution of Nox4 has been proposed in lung vessel remodeling caused by pulmonary hypertension (Barman et al., 2014). The anti-hypertensive effect of angiotensin-converting enzyme (ACE) inhibitors and AT1 receptor antagonists has been attributed in part to their ability to inhibit AngII-induced NADPH oxidase activation and expression (Schramm et al., 2012).

Endothelial nitric oxide synthase is closely associated with caveolae/lipid rafts, and critically involved in NO• production. Multiple factors influence eNOS activation, mediated through Akt-dependent phosphorylation at Ser1177, and inhibited by phosphorylation at Thr459 (Dimmeler et al., 1999). Basal eNOS activity correlates with the amount of tetrahydrobiopterin (BH4) bound to the enzyme. eNOS uncoupling occurs when there is a discrepancy between eNOS levels and NO• production, with a switch in the eNOS enzymatic activity to generate superoxide rather than NO• (Montezano and Touyz, 2012a). eNOS uncoupling is implicated in endothelial dysfunction and has been demonstrated in experimental models and patients with hypertension (essential, renovascular, malignant, and salt-sensitive), diabetes, atherosclerosis, and cardiac failure (Moens and Kass, 2007). Very limited NO• bioavailability combined with overly abundant ROS is one of the key factors causing endothelial damage and vascular dysfunction in these disorders. Moreover, classical antihypertensive agents (β-adrenergic blockers, ACE inhibitors, AT1 receptor blockers and Ca^2+^-channel blockers) exert some of their beneficial effects by stimulating NO• production and decreasing vascular ROS bioavailability (Weseler and Bast, 2010). Resveratrol, BH4, sepiapterin, and folic acid have also been shown to recouple eNOS and improve endothelial function in animal studies (Li and Forstermann, 2014).

## HHT AND OXIDATIVE STRESS

Hereditary hemorrhagic telangiectasia (HHT) is an autosomal dominant vascular dysplasia with age-related penetrance, affecting 1 in 5,000–8,000 people worldwide. The external visible signs of HHT are recurrent epistaxis (nosebleeds) and small cutaneous telangiectatic lesions. Imaging studies reveal the presence of arteriovenous malformations (AVMs)—defined as abnormal direct connections between arteries and veins—in lungs, liver, and brain. AVMs are life-threatening because of their potentially lethal complications such as stroke, hemorrhage, and brain abscess. HHT is a genetically heterogeneous disorder, HHT type 1 (HHT1) is caused by mutations in the ENG (endoglin) gene located on chromosome 9, region 9q33-q34, whereas HHT type 2 (HHT2) is caused by mutations in the ACVRL1 (activin receptor-like kinase 1 or ALK-1) gene located on chromosome 12, region 12q11-q14. About 15–20% of HHT families remain unresolved after mutation analysis of these two genes (McDonald et al., 2011), and two more genetic loci have been implicated, HHT3 on chromosome 5 (Cole et al., 2005; Govani and Shovlin, 2010) and HHT4 on chromosome 7 (Bayrak-Toydemir et al., 2006), though the causative genes remain unidentified. A subset of patients (2–3%) with a combined syndrome of juvenile polyposis and HHT (defined as JPHT) harbor mutations in the MADH4 (SMAD4) gene (Gallione et al., 2004). More recently, mutations in the BMP9 (GDF2) gene have been associated with an HHT-like syndrome. BMP9 mutations cause a vascular-anomaly syndrome with phenotypic overlap with HHT (Wooderchak-Donahue et al., 2013).

The underlying mechanism of HHT1 and HHT2 is haploinsufficiency indicating that reduced amounts of ENG and ACVRL1 lead to endothelial dysfunction and predispose to the clinical signs of disease. The observations that BMP9 is the highest affinity ligand for ACVRL1 and ENG and that Smad4 is a downstream mediator support the hypothesis that BMP9/ACVRL1/ENG/Smad4 mediated pathways are affected in HHT.

Despite the fact that vascular stress and multi-organ vascular dysplasia are hallmarks of HHT (Abdalla and Letarte, 2006), only few studies have been investigating the role of oxidative stress in the pathogenesis of this disease. We will summarize our results in mouse models of HHT, which implicate oxidative stress as an underlying mechanism of endothelial dysfunction in this disease.

### MOUSE MODELS OF HHT, ENDOTHELIAL DYSFUNCTION, AND eNOS UNCOUPLING

**FIGURE 2:**
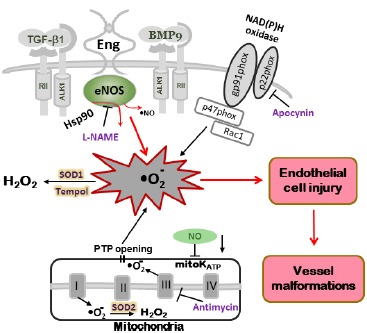
**Model of oxidative stress in mutant mice leading to vascular endothelial damage.** In *Eng*^+/–^ and *Alk1*^+/–^ mice, altered eNOS activation renders the enzyme refractory to regulation by TGF-β/BMP signaling and represents a critical event leading to excessive oxidative stress. Uncoupled eNOS produces low amounts of NO• and high levels of oxygen radicals (•O_2_^–^). The NOS inhibitor L-NAME inhibits ROS production in tissues of *Eng*^+/–^ and *Alk1*^+/–^ mice. Superoxide dismutase (SOD) and the SOD mimetic compound Tempol, converts •O_2_^–^ into less harmful hydrogen peroxide (H_2_O_2_). A large portion of ROS is produced by mitochondria (Antimycin-inhibitable) and NADPH oxidases (Apocynin-inhibitable), however, that percentage does not differ between mutant and control mice. The low NO• cellular level associated with HHT may also inhibit mitochondrial ATP-sensitive potassium channel (mitoK ATP) opening, trigger permeability transition pores (PTP) opening and further increase the oxidative stress caused by mitochondrial ROS release (adapted from Jerkic et al., 2012).

Endoglin and Acvrl1 heterozygous (*Eng*^+/–^ and *Acvrl1*^+/–^) mice have been used as models for HHT1 and HHT2 respectively, allowing us to dissect the underlying mechanisms of disease (Bourdeau et al., 1999; Srinivasan et al., 2003). Early generations of *Eng*^+/–^ mice developed multiple signs of HHT in many organs, likely due to modifier genes in the 129/Ola background (Bourdeau et al., 2001). Subsequent backcrossing to the C57BL/6J background gave rise to mice with no apparent symptoms of HHT, but allowed us to study the underlying endothelial dysfunction. Physiological studies revealed that ENG is involved in the control of vascular tone regulating NO-dependent vasodilatation (Jerkic et al., 2004). Resistance arteries from *Eng*^+/–^ C57BL/6J mice showed enhanced endothelial-dependent dilatation and impaired myogenic response (Toporsian et al., 2005). Furthermore, ENG could be found in caveolae, where it associates with eNOS and likely stabilizes it. eNOS is closely associated with caveolae/lipid rafts in endothelial cells (Pritchard et al., 2001), where ENG and ALK-1 colocalize with eNOS serving as scaffolding molecules for eNOS/Hsp90 association, eNOS activation, and NO• production (Toporsian et al., 2005). ENG-deficient endothelial cells, following Ca^2+^ activation, showed reduced eNOS/Hsp90 interaction, produced less NO• and generated more eNOS-derived superoxide (•O_2_^–^), indicating that ENG is an important regulator in the coupling of eNOS activity (Toporsian et al., 2005). Treatment of *Eng*^+/–^ resistance arteries with the anti-oxidant Tiron, reversed the vascular abnormalities (Toporsian et al., 2005), confirming that ROS are implicated in the pathobiology of HHT. Acvrl1 was also shown to associate with the eNOS activation complex leading to NO• production (Toporsian et al., 2010). Consequently, in endothelial cells deficient in ENG or Acvrl1, eNOS becomes uncoupled and produces more superoxide than NO•, leading to endothelial damage (Figure 2).

### eNOS-DERIVED ROS ARE ONLY PRESENT IN ADULT LUNGS OF *Eng* AND *Acvrl1* HETEROZYGOUS MICE

We addressed the age-dependency of ROS production in lungs of *Eng* and *Acvrl1* heterozygous mice. ROS generation was higher in adult than newborn mice, in both mutant and control mice, and so were the levels of NADPH oxidase and ROS scavenging SOD 1, 2, and 3 isoforms (Belik et al., 2009; Jerkic et al., 2011), indicating maturational changes of these enzymes. There was no difference in ROS levels between newborn mutant mice and their littermate controls, while adult mutant mice showed increased levels of pulmonary oxidative stress than control mice. Protein levels and NADPH activity were normal in adult *Eng*^+/–^ and *Acvrl1*^+/–^ lungs as well as the levels of scavenging enzymes and could not account for the increased oxidative stress. However, eNOS-dependent ROS production was increased in adult mutant mice while NO• production was reduced. Thus *Eng* and *Acvrl1* haploinsufficiency results in pulmonary vascular eNOS uncoupling in the adult, but not newborn mice (Belik et al., 2009; Jerkic et al., 2011).

Pulmonary arteries from adult *Eng*^+/–^ and *Acvrl1*^+/–^ mice were more dilated compared to the control animals. Moreover, several signs of pulmonary arterial hypertension (PAH), including elevated right ventricular systolic pressure leading to hypertrophy, reduced vascular density, and increased thickness and outward remodeling of pulmonary arterioles, were observed in adult but not newborn *Eng*^+/–^ and *Acvrl1*^+/–^ mice relative to wild-type littermate controls. Our observations support the fact that mutations in several genes of the TGF-β superfamily, including *BMPR2*, *ACVRL1*, and *ENG*, predispose to PAH (Girerd et al., 2010). Interestingly, treatment of 3-week-old *Eng*^+/–^ and *Acvrl1*^+/–^ mice with the SOD mimetic and anti-oxidant Tempol for 6 weeks prevents the onset of PAH symptoms in these mice, confirming a role for ROS in the pathobiology of the disease. These findings also suggest that an abnormal regulation of ROS might play a role in the formation of pulmonary AVMs in HHT.

### MULTIPLE ORGANS OF *Eng* AND *Acvrl1* HETEROZYGOUS MICE SHOW INCREASED ROS

Results of a more recent study (Jerkic et al., 2012) suggest that oxidative stress is indeed a generalized manifestation in organs of *Eng* and *Acvrl1* heterozygous mice. We have found increased ROS production in lungs, liver, and colon of the heterozygous mice, organs most frequently affected in HHT patients. Our results also indicate that increased oxidative stress contributes to endothelial dysfunction in mutant mice. Mitochondrial ROS production was highest in liver while NADPH oxidase was a major source of ROS in the other tissues. However, there was no difference in mitochondrial- or NADPH oxidase-dependent ROS production between mutant and control mice. In tissues of mutant mice ROS overproduction was attributed to NOS, as it was L-NAME inhibitable. As neuronal NOS was not observed and inducible NOS was barely detectable in mouse tissues (Jerkic et al., 2004, 2011), we conclude that the observed increase in ROS production in HHT mice is eNOS-derived and therefore endothelial-dependent.

A recent paper from Ghanian et al. (2014) has explored the mitochondrial redox state in kidneys and eyes of *Eng* heterozygous mice using optical imaging. They found that mitochondrial oxidative stress was decreased in these organs of *Eng*^+/–^ mice. We cannot comment on these findings, as we have not explored eye or kidney ROS production and have focused on organs most affected in HHT.

Overall, we have documented in several papers an increased oxidative stress in endothelial cells and tissues of *Eng* and *Acvrl1* heterozygous mice and identified uncoupled eNOS as a source of ROS overproduction (Figure 2). However, increased ROS concentration and decreased NO• bioavailability have further effects on ROS production and/or release from other sources, as discussed previously. Low NO• cellular levels inhibit mitoK ATP and trigger the opening of PTP. This process causes ROS release from mitochondria (Figure 2), worsening oxidative stress and leading eventually to the AVMs characteristic of HHT.

The role of oxidative stress has also been recently suggested in the pathogenesis of endothelial dysfunction in pre-eclampsia, disease associated with increased production from the placenta of a soluble form of ENG (sEng; Venkatesha et al., 2006). Tskitishvili et al. (2010) have demonstrated that cultured placental and amniotic tissues release sEng under oxidative stress, in connection with endothelial dysfunction, systemic inflammation, and pre-eclampsia.

## ANTIOXIDANTS IN CARDIOVASCULAR DISEASES

The excessive generation of ROS and/or reduced antioxidant capacity lead to endothelial dysfunction. One can therefore propose that correction of the abnormally elevated ROS bioavailability might retard, or prevent, endothelial cell injury. Antioxidants could represent a simple way to restore the redox status in the vascular milieu (Münzel et al., 2010). However, experimental and clinical studies that tested preventive or therapeutic use of antioxidants have shown that their use is much more complex and context-dependent.

Tuomilehto et al. (1987) have observed lower cardiovascular mortality in Mediterranean populations compared to those of Northern European countries and attributed the differences to the higher content of antioxidants in the Mediterranean diet. The Zutphen Elderly study confirmed a negative correlation between antioxidant vitamin intake and coronary heart disease and mortality in 805 elderly men without prior history of cardiovascular disease (Hertog et al., 1993). In line with these studies, a meta-analysis of cohort studies including almost 400,000 patients (Ye and Song, 2008) reported that high intake of antioxidant vitamins (C and E) was associated with a lower rate of coronary heart disease. However, none of the above-mentioned clinical trials directly demonstrated that the dose and type of antioxidants used effectively suppressed oxidative stress. In that context, results from the few studies that assessed antioxidant intake based on specific blood assays were more ambiguous. The ATBC study that followed 29,000 male smokers for 19 years showed that higher levels of vitamin E in the serum (but still within the physiological range) were associated with lower mortality for prostate cancer and cardiovascular disease (The Alpha Tocopherol, Beta Carotene Cancer Prevention Study Group, 1994). However, in the same study the group that received beta-carotene showed greater mortality from lung cancer. The NHANES-II study, with 8,000 enrolled subjects, showed that men but not women in the lowest quartile of serum vitamin C had an increased mortality for cancer and cardiovascular disease (Loria et al., 2000). Reasons for these equivocal and sometimes disappointing results relate to the type and dose of antioxidants used, patient cohorts, trial design, and choice of outcome measures. It is also possible that dose and duration of the antioxidant therapy were insufficient. Please refer to Hasnain and Mooradian (2004) and Stephens et al. (2009) for a detailed discussion of these findings.

Another important requirement for the clinical use of antioxidants is to develop and validate markers of oxidative stress, triage the patients with oxidative excess and measure efficacy of the treatment. It is also necessary to know the organs most affected and the major source of ROS in a particular patient, in order to choose the right antioxidant and the most efficient way for its delivery. In controlled preclinical studies and clinical trials of statins and antihypertensive drugs mentioned above, oxidative stress and NO• production were assessed. These drugs were shown to improve vascular function and reduce the incidence of cardiovascular events in patients with cardiovascular disease, at least partly by increasing NO• and reducing ROS production (Chen et al., 2008; Münzel et al., 2010; Weseler and Bast, 2010; Montezano and Touyz, 2012b).

### ANTIOXIDANTS IN HHT

We have shown using mouse models that excessive ROS generation may play a pivotal role in the pathobiology of endothelial dysfunction in HHT (Jerkic et al., 2012). Our results suggest that HHT patients may indeed benefit from diets rich in antioxidants, which could prevent or delay the rise in ROS in their blood vessels, due to eNOS uncoupling. Our studies showed that supplementing drinking water with the antioxidant Tempol for 6 weeks prevented rarefaction of the lung vasculature and the onset of PAH in *Eng* and *Acvrl1* heterozygous mice (Toporsian et al., 2010; Jerkic et al., 2011). In HHT patients, the only trial carried out to date was a pilot study in which the antioxidant *N*-acetylcysteine was given to the patients for 12 weeks (de Gussem et al., 2009). The results of this study showed beneficial effects of this antioxidant therapy with a significant decrease in the frequency and severity of epistaxis in treated HHT patients, improving their quality of life. No preventative dietary study has been carried out, although some patients with HHT have mentioned reduced nosebleeds upon maintenance on diets rich in anti-oxidants.

We propose that antioxidants are promising tools in HHT, both as preventative and therapeutic options. Prevention of endothelial dysfunction in HHT patients may include simple strategies such as an antioxidant-rich diet, abstinence from smoking, a healthier lifestyle, and regular physical activity and exercise. It has been shown that exercise can prevent eNOS uncoupling, reduce NADPH oxidase activity and decrease endothelial oxidative stress and dysfunction in experimental models and patients with coronary artery disease (Adams et al., 2005; Agarwal et al., 2011). The therapeutic use of antioxidants is much more complex and as mentioned above requires considerations of the appropriate compound and proper dosage, the affected organ, individual response to treatment, and monitoring of the therapy. The choice of the right antioxidant is most critical. We have shown that Tempol in particular is an effective antioxidant and it could be a good candidate for clinical trials in HHT patients. Tempol was successfully used in more than 70 animal studies, in diseases associated with oxidative stress, inflammation, and aging (Wilcox, 2010). Two clinical trials are presently ongoing, with the use of Tempol as a topical agent to prevent radiation-induced alopecia and for dermatitis during radiation and chemotherapy for anal cancer (http://www.paidclinicaltrials.org/dir/category/by-medicine/tt/tempol). Our experimental findings suggest that the use of antioxidants could prevent uncoupling of eNOS associated with HHT. Whether it could cause eNOS recoupling in affected organs of HHT patients remains to be tested.

In conclusion, oxidative stress plays a causative role in the pathogenesis of endothelial dysplasia and vascular malformations in HHT. The use of antioxidants in the prevention and treatment of HHT should be of benefit to the patients. More experimental and clinical studies are urgently needed to test the most effective ways to use antioxidants to ameliorate the quality of life for patients with HHT.

### Conflict of Interest Statement

The authors declare that the research was conducted in the absence of any commercial or financial relationships that could be construed as a potential conflict of interest.
